# The zinc-binding motif in tankyrases is required for the structural integrity of the catalytic ADP-ribosyltransferase domain

**DOI:** 10.1098/rsob.210365

**Published:** 2022-03-23

**Authors:** Sven T. Sowa, Lari Lehtiö

**Affiliations:** Faculty for Biochemistry and Molecular Medicine and Biocenter Oulu, University of Oulu, Oulu, Finland

**Keywords:** tankyrase, ADP-ribosyltransferase, zinc-binding motif, catalytic activity, protein stability

## Abstract

Tankyrases are ADP-ribosylating enzymes that regulate many physiological processes in the cell and are considered promising drug targets for cancer and fibrotic diseases. The catalytic ADP-ribosyltransferase domain of tankyrases contains a unique zinc-binding motif of unknown function. Recently, this motif was suggested to be involved in the catalytic activity of tankyrases. In this work, we set out to study the effect of the zinc-binding motif on the activity, stability and structure of human tankyrases. We generated mutants of human tankyrase (TNKS) 1 and TNKS2, abolishing the zinc-binding capabilities, and characterized the proteins biochemically and biophysically *in vitro*. We further generated a crystal structure of TNKS2, in which the zinc ion was oxidatively removed. Our work shows that the zinc-binding motif in tankyrases is a crucial structural element which is particularly important for the structural integrity of the acceptor site. While mutation of the motif rendered TNKS1 inactive, probably due to introduction of major structural defects, the TNKS2 mutant remained active and displayed an altered activity profile compared to the wild-type.

## Graphical abstract

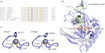

## 1. Introduction

Members of the diphtheria toxin-like ADP-ribosyltransferase (ARTD) family are widely distributed in eukaryotic organisms and serve as regulators of many processes in the cell such as DNA repair, host–virus interaction, transcription signalling and cellular stress response [1]. All 17 human ARTD members share an ADP-ribosyltransferase (ART) domain, which is responsible for catalysing the transfer of adenosine diphosphate ribosyl (ADP-ribosyl) groups. Proteins are the most common acceptors of ADP-ribosylation, but also transfer to DNA and RNA was reported for selected family members [2,3].

Tankyrases (TNKSs) catalyse the poly-ADP-ribosylation (PARylation) of their many protein targets [4–6]. While in lower animals only one TNKS exists, two highly similar TNKSs are present in humans and other vertebrates and are termed TNKS1 (PARP5a) and TNKS2 (PARP5b) [7,8]. TNKS1 and TNKS2 have overlapping functions in the cell, and it is yet unclear what the differences in molecular and physiological functions are [9,10]. Through their regulatory role in several disease-relevant pathways, TNKSs have emerged as promising drug targets, especially in the context of cancer and fibrotic diseases [11–13].

In the current model of TNKS function, TNKSs bind their substrate proteins and subsequently PARylate them, which leads to PAR-dependent ubiquitination by RNF146 and ultimately proteasomal degradation [4,14,15]. However, TNKSs also possess non-catalytic functions by acting as scaffolding proteins and mediators of protein-protein interactions, which are under investigation in current TNKS research [16–19]. Structurally, TNKSs contain five N-terminal ankyrin repeat cluster (ARC) domains. While ARC1-2 and ARC4-5 were shown to be important for binding of their various protein substrates, the ARC3 domain has likely a structural role [20,21]. A unique feature of TNKSs over other ARTD proteins is the presence of a sterile alpha motif (SAM) domain, which allows formation of large multimeric TNKS complexes [17,22–25]. The ART domain of TNKSs is located at the C-terminus and catalyses the PARylation of substrate proteins [4,26].

**Figure 1.**
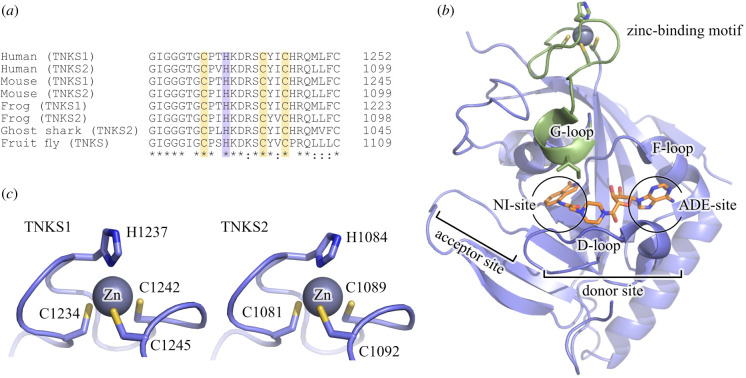
The zinc-binding motif of tankyrases. (*a*) Sequence alignment of tankyrases. The zinc-coordinating residues are highlighted. (*b*) Structure of the TNKS2 ART domain containing the zinc-binding motif (PDB: 4BJ9 [28]). The zinc-binding motif is structurally connected to the G-loop (green) located close to the acceptor site. The NAD^+^-mimic EB-47 is bound to the active site and is shown in orange. Nicotinamide sub-pocket, NI-site; adenosine sub-pocket, ADE-site. (*c*) Close-up view of the zinc-binding motif of TNKS1 (PDB: 2RF5 [26]) and TNKS2 (PDB: 4BJ9 [28]) with numbered ligand residues.

When the first crystal structure of human TNKS1 was determined, it surprisingly revealed a zinc-binding motif present in the catalytic domain located about 20 Å from the NAD^+^-binding pocket [26]. Presence of zinc in the structure of human TNKS2 was experimentally confirmed by X-ray crystallography shortly thereafter [27]. Sequence analysis shows that this motif is highly conserved among TNKSs (figure 1*a*). The zinc-binding Cys-His-Cys-Cys-type motif is located within a loop region of 12 consecutive residues in the ART domain of TNKS1 and TNKS2 (figure 1*b,c*) and does not structurally resemble known zinc-finger motifs; however, the fold can be classified as a short zinc-binding loop [29].

No other ARTD family member is known to harbour a zinc-binding function in the catalytic domain, which raised questions about its function in TNKSs. While it was speculated that it could have a structural function or be involved in regulation of catalytic activity or protein partner binding [26,30], no experimental data were available to give more insight into the function. More recently, a study by Kang *et al*. [31] found that the motif in TNKS1 may be subject to oxidative damage in the cell resulting in removal of the zinc ion and complete loss of TNKS1 activity. A direct involvement of the zinc-binding motif in the catalytic mechanism was assumed based on these findings; however, it was not discussed how exactly it would contribute to the catalysis based on the TNKS1 structure.

We were intrigued by these results, as the zinc-binding motif is distantly located (20 Å) from the active site. It is however directly connected to the G-loop, which is positioned at the nicotinamide sub-pocket at the acceptor site of TNKSs (figure 1*b*). While no residues involved in catalysis are located on the G-loop, structurally this loop may still be of importance for NAD^+^ binding and TNKS activity, in which case the zinc-binding motif might provide supporting structural role for the positioning of the G-loop.

In this work, we set out to investigate the function of the zinc-binding motif of TNKSs using biochemical, biophysical and X-ray crystallographic studies. We generated mutants in the TNKS1 and TNKS2 catalytic domains to abolish the zinc-chelating function of the respective motifs and conducted *in vitro* tests on activity and stability. We also generated a zinc-free TNKS2 catalytic domain crystal structure by oxidation of the zinc-binding motif in TNKS2 crystals, revealing structural changes occurring in the TNKS2 ART domain after removal of the zinc ion. Our results show that the zinc-binding motif is an important structural feature in TNKSs and that it is not directly required for the catalytic PARylation activity of TNKSs. However, the motif may support structures such as the G-loop region and could therefore be involved in the control and regulation of the catalytic activity.

## 2. Results

### 2.1. Design of the mutants

In all structures of the ART domain of TNKS1 and TNKS2 known to us, the electron density for zinc is clearly present, although no additional supplementation of zinc was used throughout the expression, purification and crystallization procedures of the proteins. This suggests that the zinc is bound tightly to the motif. As it has been shown that zinc-binding motifs may still retain the ability to bind zinc with lower affinity after mutation of a single ligand [32,33], we opted to introduce double mutations to abolish the zinc-binding capabilities of the motif. We generated and recombinantly produced mutants of TNKS1 and TNKS2 in which the first cysteine and the histidine of the zinc-binding motif were mutated to alanine residues. The mutated residues correspond to Cys1234 and His1237 in TNKS1, and Cys1081 and His1084 in TNKS2 (figure 1*c*). Using these mutants, the effects following the disruption of the motif and not solely the removal of the zinc ion are investigated.

### 2.2. Analysis of enzymatic activity

**Figure 2.**
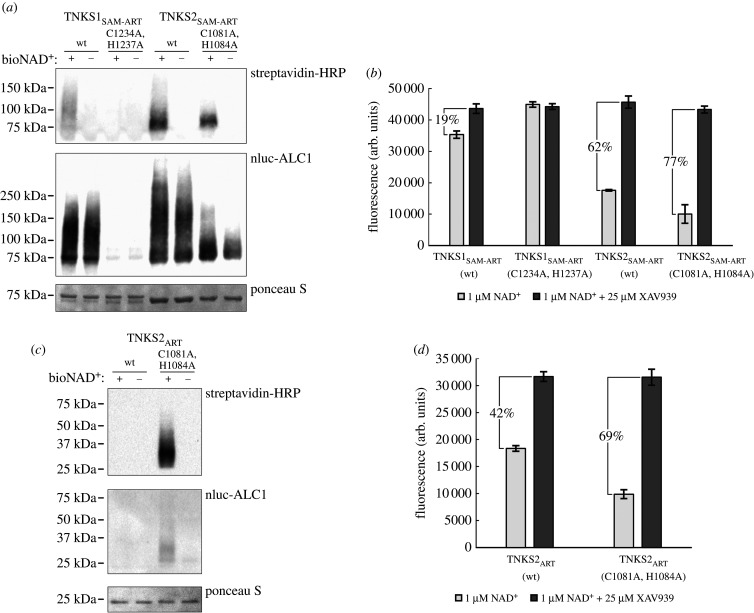
Activity of TNKS1 and TNKS2 constructs. (*a*) Western blot of TNKS1_SAM-ART_ and TNKS2_SAM-ART_ constructs tagged with maltose-binding protein (MBP). TNKS constructs (2 µM) were mixed with 100 nM biotin-NAD^+^/900 nM NAD^+^ (+) or buffer as control (−) and incubated for 1 h at room temperature. Incorporated biotin-NAD^+^ was detected with streptavidin-HRP, while PAR modification was detected using nanoluciferase-ALC1. (*b*) NAD^+^ consumption assay for MBP-tagged TNKS1_SAM-ART_ and TNKS2_SAM-ART_ constructs. The constructs (2 µM) were mixed with 1 µM NAD^+^ and incubated for 1 h at room temperature. The percentage of NAD^+^ consumption displayed was determined from the mean fluorescence values. Controls containing 25 µM XAV939 were prepared to determine fluorescence levels without consumption of NAD^+^. (*c*) Western blot of TNKS2_ART_ constructs. The constructs (2 µM) were mixed with 100 nM biotin-NAD^+^/900 nM NAD^+^ (+) or buffer as control (−) and incubated for 20 h at room temperature. The detection of PAR was done using nanoluciferase-ALC1. (*d*) NAD^+^ consumption assay for TNKS2_ART_ constructs. The constructs (2 µM) were mixed with 1 µM NAD^+^ and incubated for 20 h at room temperature. The percentage of NAD^+^ consumption displayed was determined from the mean fluorescence values. Controls containing 25 µM XAV939 were prepared to determine fluorescence levels without consumption of NAD^+^. Data shown are mean ± s.d. with number of replicates *n* = 4. Full-sized blots are shown in electronic supplementary material, figure S1.

TNKS activity was shown to be many times higher when the SAM domain present in the construct [17,23], so we tested the activities of constructs comprising the SAM and ART domains of human TNKS1 and TNKS2. All tests were done *in vitro* with recombinantly produced and purified proteins. For this, we incubated the wild-type and mutant constructs of TNKS1_SAM-ART_ and TNKS2_SAM-ART_ with biotinylated NAD^+^ analogues and detected auto-modification of the TNKS constructs by western blot with streptavidin coupled to horseradish peroxidase (HRP) (figure 2*a*). The PARylation appears as a high molecular weight smear in western blots. Similar to the report by Kang *et al*. [31], the mutation in TNKS1_SAM-ART_ completely abolished auto-modification levels compared to the wild-type. Interestingly, the mutated TNKS2_SAM-ART_ retained auto-modification activity; however, the smear seems to be visible only in lower molecular weight compared to the wild-type, possibly indicating formation of shorter PAR chains or modification of fewer acceptor sites. We also visualized total levels of PARylation with the PAR-binder ALC1 fused to nanoluciferase [34]. Similarly here, PARylation smears are detected for all constructs except for the TNKS1 mutant. The controls containing no NAD^+^ during the reaction showed also high PARylation levels, which have occurred during expression in *Escherichia coli* (figure 2*a*).

In addition to auto-modification, NAD^+^ may also be converted directly by hydrolysis, as several ARTs were reported to have NAD^+^ hydrolase activity [35,36]. To test the total combined NAD^+^ consumption from both PARylation and direct hydrolysis, we used an assay based on chemical conversion of NAD^+^ into a fluorescent compound [37]. (Figure 2*b*). Again, only the mutant of TNKS1_SAM-ART_ did not show any consumption of NAD^+^. Interestingly, despite seemingly lower auto-PARylation activity, the TNKS2_SAM-ART_ mutant showed very similar and possibly higher levels of NAD^+^-consumption compared to the wild-type.

Because the SAM domain is an important determinant for TNKS activity, we wanted to investigate the impact of the zinc-binding mutant on the activity in absence of the SAM domain. For this, we similarly to the above tested the auto-PARylation (figure 2*c*) and total NAD^+^ consumption (figure 2*d*) for the catalytic domain of TNKS2 and compared wild-type and mutant. Due to the lower activity in absence of the SAM domain, a 20-fold longer incubation time was necessary to achieve similar NAD^+^ consumption levels. Interestingly, while auto-PARylation of the wild-type catalytic domain could not be detected, a clear smear was present for the zinc-binding mutant. Further, total NAD^+^ consumption was determined to be 69% for the mutant. For the wild-type, a lower NAD^+^ consumption of only 42% was measured. Both TNKS2_ART_ constructs bind and hydrolyse NAD^+^ as shown by the total NAD^+^ consumption. However, it appears the zinc-binding mutant of TNKS2_ART_ more readily transfers ADP-ribosyl groups to protein substrates, at least in the context of auto-modification. A possible explanation may be a structural change in the zinc-binding mutant, allowing accepting protein residues to adopt a more favourable orientation and proximity for the transfer of ADP-ribose. A similar and more pronounced change may occur in the presence of the SAM domain upon multimerization, which could explain why such difference in ADP-ribosylation activity between wild-type and mutant constructs of TNKS2_SAM-ART_ constructs was not observed.

These results indicate that the activity of TNKS2 is affected upon mutation of the zinc-binding motif, but that the presence of zinc is not required for binding of NAD^+^ and catalysing ADP-ribosylation. However, why do we observe complete abolishment of activity in the TNKS1_SAM-ART_ mutant, while the TNKS2_SAM-ART_ mutant shows comparable or even higher levels of NAD^+^ consumption compared to the wild-type? A possible explanation might be improper folding of TNKS1 upon introduction of the mutation. We therefore set out to test the thermal stability of the produced TNKS1 and TNKS2 proteins.

### 2.3. Thermal stability and folding analysis

**Figure 3.**
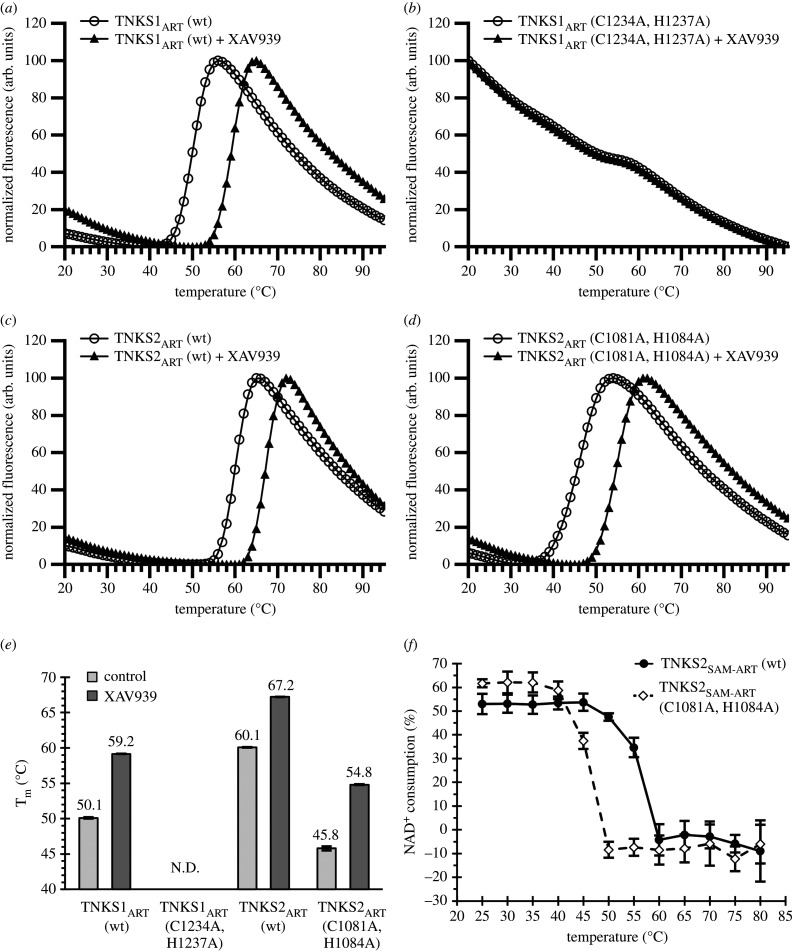
Thermal stability of TNKS1 and TNKS2 constructs. Representative DSF curves in presence or absence of 25 µM XAV939 for (*a*) TNKS1_ART_ wild-type, (*b*) TNKS1_ART_ zinc-binding mutant, (*c*) TNKS2_ART_ wild-type and (*d*) TNKS2_ART_ zinc-binding mutant. (*e*) Comparison of melting temperatures determined from four independent DSF curves for each condition. Mean melting temperature (T_m_) values are indicated, and errors are shown as s.d. Melting temperatures for TNKS1_ART_ could not be determined. (*f*) NAD^+^ consumption assay for MBP-tagged TNKS2_SAM-ART_ samples (2 µM) after incubation at different temperatures. Data shown are mean ± s.d. with number of replicates *n* = 4.

To test the thermal stability by differential scanning fluorimetry (DSF), we used constructs containing only the ART domain of TNKS1 and TNKS2 both wild-type and zinc-binding mutants as the unfolding of additional SAM domain and possibly formation of multimeric complexes would interfere with the analysis. We performed DSF analysis with all four constructs in absence or presence of TNKS inhibitor XAV939 (figure 3*a–e*). The melting temperature (T_m_) of TNKS1_ART_ wild-type was determined to be 50.1°C (figure 3*a*). Analysis of the TNKS1_ART_ mutant however showed an initially high fluorescence value, which decreased as the sample temperature increased with no clear inflection point (figure 3*b*). As a result, the T_m_ for the TNKS1_ART_ mutant could not be determined. This behaviour in DSF-based experiments is associated with disordered, aggregated or incorrectly folded proteins [38]. Compared to TNKS1_ART_, the TNKS2_ART_ wild-type showed higher thermal stability of 60.1°C (figure 3*c*), while the zinc-binding mutant showed a dramatic decrease in thermal stability of about 14°C with a T_m_ of 45.8°C (figure 3*d*). The wild-type of TNKS1_ART_ and TNKS2_ART_ as well as the TNKS2_ART_ zinc-binding mutant showed an increased thermal stability of approximately 7–9°C when the potent TNKS inhibitor XAV939 was added, indicating the binding of the molecule. By contrast, the curve from the zinc-binding mutant of TNKS1_ART_ did not show changes with XAV939 added. The melting temperatures determined are summarized in figure 3*e*.

To see if a strong loss of stability in the TNKS2 mutant could also be observed in multimeric TNKS2, we incubated wild-type and mutant TNKS2_SAM-ART_ constructs at different temperatures ranging from 20°C to 85°C for 1 min per sample (figure 3*f*). After incubation, the protein samples were mixed with NAD^+^, and activity was determined using the NAD^+^ consumption assay described above. The complete loss of activity is seen for the mutant construct after incubation at 50°C, while the wild-type still retained most of its activity after incubation at this temperature. Only after incubation at 60°C did the wild-type show complete loss of activity. These results indicate that the zinc-binding motif is a stabilizing structural feature in the case of TNKS2 for both the isolated ART domain and the multimeric construct containing the SAM and ART domains.

**Figure 4.**
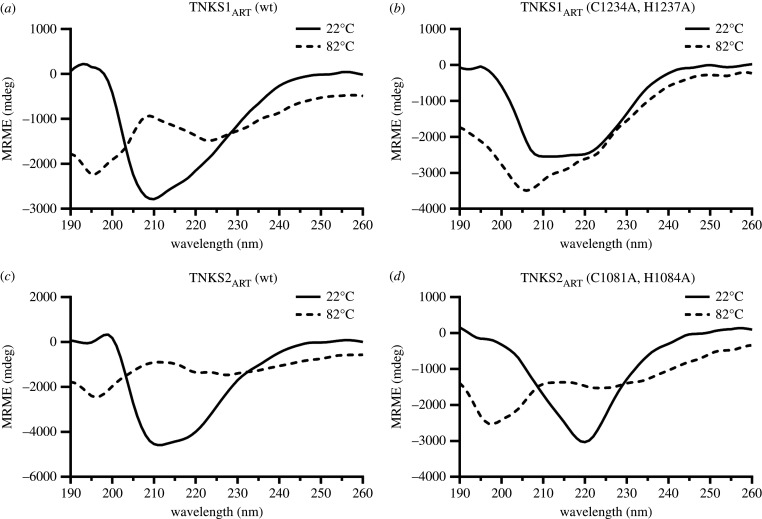
CD spectra of catalytic domain constructs of TNKS1 and TNKS2. The CD spectra were recorded from 190 nm to 260 nm at 22°C and 82°C for the ART domains of (*a*) TNKS1_ART_ wild-type, (*b*) TNKS1_ART_ zinc-binding mutant, (*c*) TNKS2_ART_ wild-type and (*d*) TNKS2_ART_ zinc-binding mutant. CD spectra for all temperatures are shown in electronic supplementary material, figure S2.

To further characterize possible structural changes introduced by the mutation of the zinc-binding motif in TNKS1 and TNKS2 ART domains, we analysed the TNKS_ART_ constructs using circular dichroism (CD) spectroscopy. Samples were heated from 22°C to 82°C, and CD spectra from 190 nm to 260 nm were determined in 2°C intervals. CD spectra at 22°C and 82°C are shown in figure 4*a–d*. Spectra for all temperatures are shown in electronic supplementary material, figure S2. The CD spectrum of the TNKS1_ART_ wild-type construct (figure 4*a*) at 22°C shows a minimum at 208 nm, which inverts to a maximum after heating to 82°C. Additionally, minima at 195 nm and 222 nm appear, indicating the thermal unfolding of the protein [39]. By contrast, the TNKS1_ART_ mutant (figure 4*b*) at 22°C additionally shows a minimum at 220 nm. After heating to 82°C, no inversion of minima is observed, with only the initial minimum at 208 nm gradually transitioning to 205 nm with no clear inflection point as the temperature increased (electronic supplementary material, figure S2). The spectra of the TNKS2_ART_ wild-type (figure 4*c*) strongly resemble those of the TNKS1_ART_ wild-type, with a more pronounced shoulder at 220 nm at 22°C. Interestingly, a major difference in the spectrum is observed at 22°C for the TNKS2_ART_ mutant (figure 4*d*) compared to the wild-type. While no minimum at 208 nm is observed, the construct shows a clear minimum at 220 nm. When heated, the spectrum transitioned comparable to TNKS1_ART_ and TNKS2_ART_ wild-type constructs with minima around 195 and 222 nm, a maximum at around 210 nm and a clear inflection point (electronic supplementary material, figure S2). These results indicate that mutation of the zinc-binding motif introduces structural changes in the ART domains of TNKS1 and TNKS2. In the case of TNKS1, when considering the DSF result associated with unstructured proteins and only a small gradual change of the CD spectrum upon heating, we believe that mutation of the zinc-binding motif in TNKS1 leads to misfolding of the protein, which likely explains the lack of activity observed in figure 2*a*,*b*. Interestingly, the mutation of the zinc-binding motif in TNKS2 leaves the protein active, and it is still stabilized by the specific TNKS inhibitor XAV939 in DSF, indicating the presence of a functional binding pocket. Compared to the wild-type however, the TNKS2 mutant is severely less thermostable, and comparison of the CD spectra at 22°C indicates structural changes, which might explain the different behaviour in catalytic activity observed when compared to the wild-type.

### 2.4. Structural analysis

**Figure 5.**
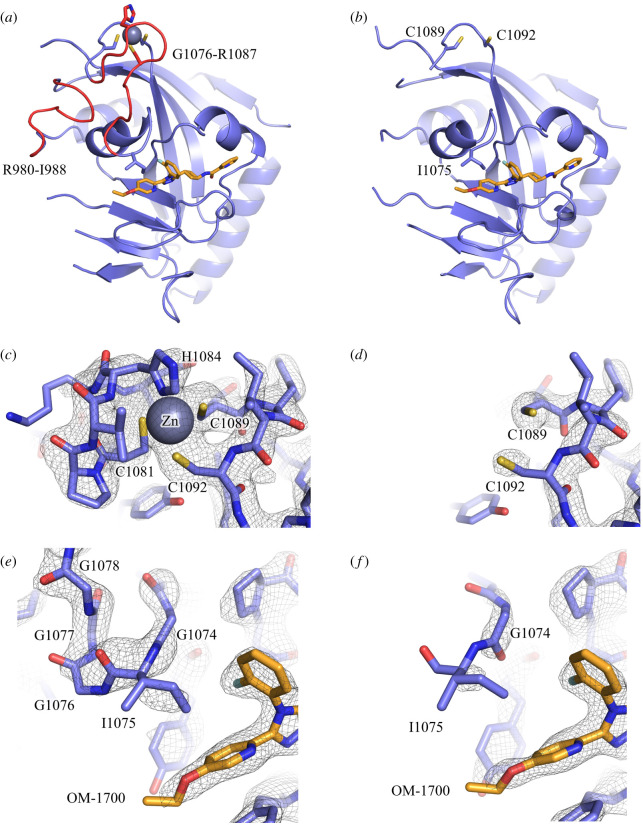
Structural changes in the TNKS2 ART domain upon oxidative removal of zinc. (*a*) Crystal structure of human TNKS2 ART domain in complex with OM-1700 (PDB ID: 6TG4 [41]). Red parts of the protein structure show regions (R980-I988, G1076-R1087) that were not possible to model after H_2_O_2_ treatment. (*b*) Human TNKS2 ART domain in complex with OM-1700 after the crystals were treated for 48 h with 25 mM H_2_O_2_ (PDB ID: 7POX). The zinc-binding motif region is shown for the structures without (*c*) and after (*d*) H_2_O_2_ treatment. A closeup view of the nicotinamide sub-pocket with the bound inhibitor OM-1700 (orange) without (*e*) and after (*f*) H_2_O_2_ treatment. The *σ*_A_ weighted 2F_o_ – F_c_ electron density maps are contoured at 1.6 *σ*.

We aimed to get insight into structural changes occurring in TNKS2 upon disruption of the zinc-binding motif by protein X-ray crystallographic studies. Initial crystallization trials with the zinc-binding mutant of the TNKS2 ART domain remained unsuccessful, which we find not surprising considering the dramatically lower thermal stability compared to the wild-type. As alternative strategy, we crystallized the wild-type of the TNKS2 ART domain and attempted to remove the zinc from the crystals. Soaking the crystals for 48 h in 25 mM EDTA, a potent chelator of zinc ions, did not show any visible loss of zinc in the structure. We next opted to oxidize the cysteine residues with H_2_O_2_ after protein crystal formation. First attempts with the apo-protein of TNKS2 revealed complete loss of diffraction after H_2_O_2_ treatment, possibly through introduced disorder in the crystal lattice. A different crystal system with TNKS2 can be achieved when co-crystallizing it with inhibitors binding to the adenosine sub-pocket [40,41]. Treatment with H_2_O_2_ of TNKS2 crystals that were co-crystallized with our previously reported TNKS inhibitor OM-1700 did not significantly lower diffraction quality. While lower concentrations of H_2_O_2_ showed only partial loss of the zinc ion, incubation of the crystals with 25 mM H_2_O_2_ for 48 h showed complete loss of electron density corresponding to the zinc ion in the crystal structure. When compared to the structure of non-treated crystals (figure 5*a*), the structure of H_2_O_2_ treated crystals (figure 5*b*) showed loss of electron density for the zinc ion (figure 5*c,d*) and of several protein regions nearby.

Interestingly, residues Gly1076-Arg1087 connecting from the zinc-binding motif up to the Ile1075 located in the G-loop could not be modelled after H_2_O_2_ treatment, indicating increased flexibility of this region (figure 5*e*,*f*). Although many of residues in this region are glycines, they form a helical structural element with a defined hydrogen bonding network in TNKSs when the zinc-binding motif is intact (electronic supplementary material, figure S3). Additionally, the residues Arg980-Ile988 also showed loss of electron density in chain A of the H_2_O_2_-treated sample (electronic supplementary material, figure S4). This region is located close to the zinc-binding motif and under native conditions is connected by hydrogen bonds to Ser1088 and Arg1087. Electron density for this region was present in chain B, likely due to nearby crystal contacts preventing flexibility. Apart from these unmodelled regions connected to the zinc-binding motif, the modelled regions of the two structures showed no obvious differences (root-mean-square deviation = 0.75 Å).

## 3. Discussion

Based on findings from Kang *et al*. [31], it was assumed that the zinc-binding motif would be required for the catalytic activity of TNKSs. Our work shows that the motif is not a strict requirement for the catalytic activity, as PARylation and NAD^+^ consumption were clearly observed for both TNKS2_SAM-ART_ and TNKS2_ART_ mutant constructs. For TNKS1, the activity of the mutant construct was greatly reduced or abolished. Experiments using DSF and CD however showed evidence for incorrectly folded TNKS1 zinc-free mutant protein, whereas the mutant of TNKS2 showed presence of a folded domain, although it had drastically reduced thermal stability. Kang *et al*. [31] observed complete loss of TNKS1 activity when mutants abolishing the zinc-binding function were used or the zinc was oxidatively removed. Following these findings, they concluded that the zinc-binding motif in TNKSs may be catalytically essential. However, our results indicate that the TNKS structure (but not directly the catalytic mechanism itself) is affected upon removal of the zinc, which in turn may lead to structural defects in TNKS1 causing the loss in catalytic activity.

Analysis of the TNKS2 structure after oxidative removal of the zinc ion revealed missing election densities for regions Gly1076-Arg1087 and Arg980-Ile988, likely due to increased flexibility in these regions following the disruption of the zinc-binding motif. This loss of structure at the G-loop, which is located at the active site nicotinamide sub-pocket of TNKSs, might affect the activity and can be taken as a possible explanation for the different activities observed in TNKS2 zinc-binding mutant constructs compared to the wild-type proteins.

TNKS ART domains have unique structural adaptations compared to other ARTD family members. These include the zinc-binding motif and the structurally connected G-loop protruding into the active site and forming part of the hydrophobic ‘nook’ in TNKSs. The unique makeup of this region benefitted drug discovery efforts and allowed development of several highly specific TNKS inhibitors [4,42]. Why do TNKSs have these adaptations compared to other family members? Examining the molecular functions that are unique to the TNKSs might give clues about why these features are present in the TNKS ART domains.

TNKSs and PARP1 and PARP2 are the only ARTD family members in humans that have reported PARylation activity [43]; however, TNKSs are evolutionarily more closely related to many of the mono-ADP-ribosylating family members [44]. PARylation activity of TNKSs might have evolved independently from PARP1 and PARP2 and thus might have different underlying structural adaptations for the synthesis of PAR chains. In fact, the PAR chains produced by TNKSs were reported to be linear[5], while PARP1 and PARP2 are able to produce branched PAR chains [45,46]. This was reasoned to be due to a more restrained acceptor site of TNKSs compared to PARP1 and PARP2, limiting the orientation of a possible acceptor-ADP-ribosyl moiety [4]. In our western blot experiments, we observed that smears of auto-modified TNKS2_SAM-ART_ zinc-free mutant were running at lower molecular weights compared to the wild-type construct, indicating the formation of shorter PAR chains. In PARP1, the acceptor site is required for binding of accepting ADP-ribosyl or PAR moieties during chain elongation [47]. Assuming the same would be true for TNKSs, an intact zinc-binding region may be required for proper formation of long PAR chains.

Another feature of TNKSs is the upregulation of catalytic activity upon multimerization by the SAM domain. It was proposed that a conformational change in the TNKS ART domains might occur through formation of weak ART domain dimers upon SAM-dependent multimerization [48], however more work and structural insights are required to confirm this mechanism. We have observed that both mutant and wild-type TNKS2_SAM-ART_ constructs show drastically higher levels of NAD^+^ consumption *in vitro* compared to the TNKS2_ART_ constructs, indicating that the upregulation of activity upon SAM-dependent multimerization is still present when the zinc-binding motif is removed. We note however that the zinc-binding mutant in the TNKS2_ART_ construct showed much higher levels of auto-modification and NAD^+^ consumption compared to the wild-type.

Furthermore, the ability of TNKSs to bind a plethora of different proteins through their ARC domains and subsequent modification of these proteins is a feature that might require structural adaptations in the makeup of the ART domain. Based on our results, we cannot exclude the possibility that the zinc-binding motif and structurally connected regions play a role in this function or the binding of other yet unknown partners.

In summary, our analysis has revealed that the zinc-binding motif in TNKSs is an important structural feature of the TNKS ART domain and is tightly linked to the structural integrity of the protein and particularly the acceptor site region. Comparing the wild-type and zinc-binding mutant, we observed differences in the PARylation activity of TNKS2, which may be indicative of a role for the zinc-binding motif in catalytic activity regulation, probably through supporting of structural features in the vicinity of the active site. Our findings contribute to the understanding of the structural makeup of the catalytic domains of TNKSs, and may help to characterize the basis for TNKS activity control and regulation in the future.

## 4. Material and methods

### 4.1. Sequence analysis

TNKS sequences were collected from the UniProt database [49]: *Homo sapiens* TNKS1 (O95271), *H. sapiens* TNKS2 (Q9H2K2), *Mus musculus* TNKS1 (Q6PFX9), *M. musculus* TNKS2 (Q3UES3), *Xenopus laevis* TNKS1 (A0A1L8HN28), *X. laevis* TNKS2 (A0A1L8FES1), *Callorhinchus milii* TNKS2 (A0A4W3JSL5) and *Drosophila melanogaster* TNKS (Q9VBP3). The sequences were aligned using Clustal Omega [50].

### 4.2. Cloning

Inserts of the fusion constructs were prepared by PCR: TNKS1_ART_ (1091–1327), TNKS1_SAM-ART_ (1017–1327), TNKS2_ART_ (946–1161) and TNKS2_SAM-ART_ (Met-873–1161). Double mutations of the zinc-binding motif (TNKS1: C1234A, H1237A; TNKS2: C1081A, H1084A) were introduced by overlap-extension PCR using the constructs above as templates. The expression constructs were cloned into pNIC-MBP plasmids using the sequence and ligation independent cloning (SLIC) method [51] as previously described [19].

### 4.3. Protein expression

Expression constructs are based on pNIC-MBP and contain a His_6_-MBP-tag followed by a TEV protease cleavage site (ENLYFQ*SM) before the construct sequence. *Escherichia coli* BL21(DE3) cells were transformed with the plasmids. Five-hundred millilitres of terrific broth autoinduction media including trace elements (Formedium, Hunstanton, Norfolk, England) were supplemented with 8 g l^−1^ glycerol and 50 µg ml^−1^ kanamycin, and inoculated with 5 ml of overnight preculture. The flasks were incubated shaking at 37°C until an OD_600_ of 1 was reached. The temperature was set to 18°C and incubation continued overnight. The cells were collected by centrifugation at 4200*g* for 30 min at 4°C. The pellets were resuspended in lysis buffer (50 mM HEPES pH 7.5, 500 mM NaCl, 15 mM imidazole, 0.5 mM TCEP). Cells were stored at −20°C until purification.

### 4.4. Protein purification

All constructs were initially purified by immobilized metal affinity chromatography followed by purification on an MBPTrap HP column (Merck). The cells were thawed and lysed by sonication. The lysate was centrifuged (16 000*g*, 4°C, 30 min), filtered and loaded onto a 5 ml HiTrap HP column (Merck) equilibrated with lysis buffer and charged with Ni^2+^. The column was washed with five column volumes of lysis buffer and five column volumes of lysis buffer containing 25 mM imidazole. The protein was eluted using lysis buffer containing 300 mM imidazole. Elutions were directly loaded to a 5 ml MBPTrap HP column. The column was washed with five column volumes 20 mM HEPES pH 7.5, 200 mM NaCl, 0.5 mM TCEP and eluted using the same buffer including 10 mM maltose. The elutions of TNKS1_ART_ and TNKS2_ART_ constructs were treated with TEV protease (1 : 30 molar ratio) for 16–20 h at 4°C. The buffer was exchanged to 20 mM HEPES pH 7.5, 50 mM NaCl, 10% glycerol and 0.5 mM TCEP, and the protein was loaded to a 5 ml HiTrap SP FF cation exchanger column (Merck). The buffer used was 20 mM HEPES pH 7.5, 10% glycerol and 0.5 mM TCEP, and a linear gradient to a final concentration of 500 mM NaCl over 60 ml was used to elute the protein. To remove residual MBP present, the eluted protein was run over a 5 ml MBPTrap HP column equilibrated with 20 mM HEPES pH 7.5, 500 mM NaCl, 0.5 mM TCEP and 10% glycerol. Proteins were concentrated, frozen in liquid nitrogen and stored at −70°C.

### 4.5. Western blot

Reactions of the protein constructs and NAD^+^ were done in 50 mM Bis-Tris-Propane pH 7.0, 0.01% Triton X-100, 0.5 mM TCEP. For western blot, 10 µl samples were first run in SDS-PAGE (Mini-Protean TGX 4–20% gradient gel, BioRad). The proteins were then transferred to a nitrocellulose membrane using a Mini Trans-Blot Cell Wet/Tank blotting system (BioRad). After transfer, membranes were washed in TBS-T (tris-buffered saline containing 0.1% Tween 20) and stained using Ponceau S solution and imaged. Staining was removed by washing the membrane in TBS-T, and the membrane was blocked for 20 min using 1% Casein in TBS (BioRad). To detect PAR chains with incorporated 6-Biotin-17-NAD^+^ (Biolog), the membrane was transferred to 15 ml blocking solution including streptavidin-HRP (1:5000) and incubated for 20 min. The membrane was washed using TBS-T buffer, covered with enhanced chemiluminescence (ECL) substrate solution (BioRad) and imaged using a ChemiDoc Imaging System (BioRad). The blot was washed with TBS-T and TBS-T including 5% skimmed milk powder. To detect PAR groups, the membrane was transferred to 15 ml TBS-T including 1% skimmed milk powder and nanoluciferase-ALC1 (0.1 µg ml^−1^) [34] and incubated for 20 min. The membrane was washed with TBS-T and imaged using 500 µl of 1 : 500 NanoGlo substrate (Promega, catalogue no. N1120) diluted in 10 mM sodium phosphate buffer pH 7.0.

### 4.6. NAD^+^ consumption assay

The NAD^+^ consumption assay is based on our previously described method [37]. All reactions were prepared in 50 mM Bis-Tris-Propane pH 7.0, 0.01% Triton X-100, 0.5 mM TCEP. Briefly, 5 µl reactions were prepared in 384-well ShallowWell black polypropylene plates (Fisherbrand). TNKS_SAM-ART_ or TNKS_ART_ constructs (2 µM) were mixed with 1 µM NAD^+^ and let incubate at room temperature for 1 h or 20 h, respectively. Controls containing 25 µM XAV939 preventing the NAD^+^ consumption were prepared and used for the calculation of the NAD^+^ consumption percentage. The reactions were stopped by addition of 1 µl KOH (2 M) and 1 µl ethanol containing 10%(v/v) acetophenone and 30%(v/v) glycerol. The mixtures were incubated for 10 min at room temperature. Finally, 3 µl formic acid (100%) were added, and the reactions incubated for 5 min at room temperature. The fluorescence was read using a Spark multimode plate reader (Tecan) with excitation wavelength of 372 nm (10 nm bandwidth) and emission wavelength of 444 nm (20 nm bandwidth). The reactions were prepared using a Mantis microfluidic liquid handler (Formulatrix).

### 4.7. Differential scanning fluorimetry

All samples of TNKS1_ART_ or TNKS2_ART_ constructs were prepared in 20 mM HEPES pH 7.5, 500 mM NaCl, 0.5 mM TCEP and 10% glycerol. The constructs (5 µM) were mixed with SYPRO orange (2×) in presence of absence of 25 µM XAV939. Samples were transferred to 96-well quantitative PCR plates. Measurements were performed in a BioRad C1000 CFX96 thermal cycler. Data points for melting curves were recorded in 1 min intervals from 20°C to 95°C, with the temperature increasing by 1°C min^−1^. The analysis of the data was done in GraphPad Prism 7 using a nonlinear regression analysis (Boltzmann sigmoid equation) of normalized data.

### 4.8. Circular dichroism spectroscopy

TNKS1_ART_ or TNKS2_ART_ constructs were diluted to 20 µg ml^−1^ in 10 mM sodium phosphate buffer (pH 7.5). CD spectra were recorded using a Chirascan CD Spectrometer (Applied Photophysics). Spectra (190 to 260 nm) were recorded in 2°C intervals from 22°C to 82°C, with the temperature increasing by 1°C min^−1^.

### 4.9. Crystallography and data collection

Protein crystallization for TNKS2_ART_ in complex with OM-1700 was done as previously described [41]. Protein crystals were soaked in reservoir solution containing 25 mM H_2_O_2_ for 48 h. Data were collected at Diamond Light Source (DLS) on beamline i03. Diffraction data were processed using the XDS package [52]. All structures were solved using molecular replacement with PHASER [53] using the structure of TNKS2 (PDB code: 5NOB) as a starting model. Coot [54] and Refmac5 [55] were used for model building and refinement, respectively. The images of the structures were prepared using PyMOL (The PyMOL Molecular Graphics System, v. 1.8.4.0, Schrödinger). Data collection and refinement statistics are shown in electronic supplementary material, table S1.

### 4.10. Calculation of the root-mean-square deviation

Calculation of the root-mean-square deviation values was done using PyMOL (The PyMOL Molecular Graphics System, v. 1.8.4.0, Schrödinger). Alignment was performed without outlier rejection using the command ‘align object1, object2, cycles = 0, transform = 0’. Chain A of TNKS2 in complex with OM-1700 after H_2_O_2_ treatment (PDB: 7POX) was aligned against the corresponding chain of the TNKS2 structure in complex with OM-1700 without treatment of H_2_O_2_ (PDB: 6TG4). A root-mean-square deviation of 0.75 Å was calculated from 1455 aligned atoms.

## Data Availability

Atomic coordinates and structure factors have been deposited to the Protein Data Bank (PDB) under accession number 7POX, and raw diffraction images are available at IDA (https://doi.org/10.23729/16748d49-b84c-4114-9431-ecd8ae615623).
